# Analysis of the current situation and influencing factors of emotional labor among pediatric nurses: A cross-sectional study

**DOI:** 10.1371/journal.pone.0347760

**Published:** 2026-04-24

**Authors:** Huiqing Liu, Zifeng Li, Xiaojing Zhou, Wei Gong, Zuyang Xi, Juan Cao

**Affiliations:** 1 The First College of Clinical Medical Science, Three Gorges University/Yichang Central People’s Hospital, Yichang, Hubei Province, China; 2 Jiangxi Provincial Children’s Hospital/The Affiliated Children’s Hospital of Nanchang Medical College, Nanchang City, Jiangxi Province, China; STIKES Wira Medika PPNI Bali: Sekolah Tinggi Ilmu Kesehatan Wira Medika PPNI Bali, INDONESIA

## Abstract

**Aim:**

To examine the level and factors associated with emotional labor among pediatric nurses in Yichang, China.

**Methods:**

From December 20–25, 2024, a cross-sectional descriptive online study was conducted among 307 pediatric nurses in Yichang, China. The survey included general information, emotional labor (assessed using the Emotional Labor Scale; higher scores indicate greater emotional labor), spiritual climate, and compassion satisfaction, and the influencing factors associated with emotional labor were analyzed.

**Results:**

Pediatric nurses in Yichang had a total emotional labor score of 69.20 ± 9.40. Regression analysis showed that nurses without children had higher emotional labor levels than those with children (P < 0.05). Higher spiritual climate and compassion satisfaction scores were both significantly associated with higher emotional labor scores (P < 0.05).

**Conclusions:**

The intensity of emotional labor among pediatric nurses in Yichang is high and is associated with parental status (having children), spiritual climate, and compassion satisfaction.

## 1. Introduction

Emotional labor was originally proposed by the sociologist Hochschild. It refers to the process by which individuals regulate and manage their emotions to meet organizational goals or occupational requirements [1–2]. In medical settings, nurses’ emotional labor is particularly pronounced, as manifested in their interactions with patients and their families. Nurses are expected to display compassion, patience, caring, and other positive emotions. They must maintain a professional attitude even when facing stress, fatigue, or negative emotions [3–4]. This regulation involves not only emotional expression through surface acting (SA) but also deeper emotional regulation through deep acting (DA). Surface acting (SA) entails faking emotions without changing naturally felt states, whereas deep acting (DA) involves cultivating and expressing genuine emotions in accordance with social and organizational norms [5].

Pediatric nursing amplifies these demands. Most pediatric patients cannot express themselves clearly, while their families often hold high expectations for care; as a result, pediatric nurses invest more emotional effort [6]. They routinely face ill children and anxious family members, creating greater emotional stress and necessitating a higher frequency and intensity of emotional labor than in other units [7].

Emotional labor has dual effects. Positively, displaying care, patience, and compassion can increase patient satisfaction and trust. Relief of pain or improved mood can lead to professional achievement and satisfaction, reinforcing identity and motivation [8–10]. Negatively, sustained emotional labor can harm physical and mental health. Adverse outcomes include emotional exhaustion, burnout, and mental health problems [11]. Over time, emotional labor depletes emotional resources and contributes to burnout, manifesting as numbness, reduced job satisfaction, and heightened turnover intentions [12–16]. It is also linked to anxiety and depression, impairing performance and quality of life [17–19].

Within the Conservation of Resources (COR) theory, emotional labor reflects the expenditure of emotional and psychological resources to meet job demands [20,21]. Individuals seek to acquire, maintain, and protect resources, particularly when threats or depletion outpace replenishment. This pattern is pronounced in pediatric nursing, marked by stress, fatigue, and burnout [22,23]. Resource exhaustion is closely tied to work settings: confronting the pain of fragile children, communicating with anxious families, and frequently navigating high-risk situations. Determinants span individual and environmental domains. Emotion regulation capacity and psychological resilience function as personal resources that conserve and replenish emotional reserves [24,25]. Excessive workload accelerates consumption, whereas organizational support and a positive nurse–patient relationship buffer pressure, alleviate fatigue, and promote recovery [26,27].

However, in pediatric nursing, within the Conservation of Resources (COR) theoretical framework, there remains a lack of clarity about how spiritual climate and compassion satisfaction, as key antecedent variables, relate to nurses’ emotional labor. Spiritual climate refers to a supportive workplace atmosphere formed through collaboration, communication, trust-building, and the sharing of inner experiences among individuals and team members [28]. As an important situational resource, it may be associated with enhanced psychological resilience and with buffering against the adverse effects linked to emotional labor. Compassion satisfaction is defined as the positive affect derived from helping others, typically manifesting as a sense of meaning and enjoyment obtained from caregiving work [29]. It is a key personal resource/gain arising from the emotional rewards of successfully helping patients and is associated with offsetting emotional exhaustion linked to emotional labor. Furthermore, based on COR theory, this study posits that parenting experience may likewise constitute an important personal resource; its presence or absence may be associated with nurses’ subjective experience of emotional labor and with their regulation strategies, whereby nurses possessing this resource may be better positioned to meet the emotional demands encountered in pediatric care.

Despite growing attention to emotional labor in nursing, pediatric nursing remains understudied, especially under the high-pressure conditions of Chinese public hospitals. Prior work largely centers on general nurses, overlooking the distinct emotional demands of pediatric care. Using a cross-sectional descriptive survey, we assessed the status and influencing factors of emotional labor among pediatric nurses in Yichang, addressing gaps in previous studies. We quantified the strength of correlations between demographic factors, spiritual climate, compassion satisfaction, and emotional labor, providing a foundation for targeted interventions.

## 2. Materials and methods

### 2.1. Study design and setting

This study was a descriptive cross-sectional study of pediatric nurses in 15 general hospitals with pediatric wards in Yichang, Hubei Province, China.

### 2.2. Participants

Pediatric nurses from 15 public hospitals in Yichang were recruited using convenience sampling. Inclusion criteria were: (1) nurses with practicing qualification certificates and (2) at least one year of experience, currently working in pediatrics. Nurses were excluded if they were on leave, engaged in advanced studies, interns, or assistant nurses. Sample size was determined using the empirical rule for regression analysis, ensuring 10−20 samples per independent variable [30]. With 12 independent variables, and accounting for a 20% rate of invalid questionnaires, the minimum sample size calculated was n = (12*20)/(1–0.2) = 300. Ultimately, 314 questionnaires were collected. After excluding 7 patterned answer sheets, 307 valid samples remained, yielding an effective response rate of 97.77%.

### 2.3. Data collection

Data was collected online from December 20–25, 2024. The study team collaborated with the administrative offices of the participating hospitals to disseminate the survey link to eligible individuals through dedicated WeChat groups. Upon accessing the online link, participants were presented with an introductory page outlining the study objectives, instructions on the questionnaire, confidentiality statements, and an electronic consent form. Only those who confirmed consent were directed to the main questionnaire. All participants completed the questionnaire using their mobile phones or personal computers. The questionnaire required completion of all items for submission, and duplicate submissions were minimized by restricting each participant to a unique survey link. The survey link was accessible only to invited users to prevent unauthorized sharing, and the survey was conducted anonymously, ensuring no personal privacy was leaked. Data entry was completed by two researchers: one responsible for data output and statistical analysis, and the other for repeated analysis and proofreading of the data pairs to ensure data accuracy and reliability.

### 2.4. Ethical considerations

This study complied with the Declaration of Helsinki and was approved by the Ethics Committee of Yichang Central People’s Hospital (Ethics No. 2024-525-01). The Chinese version of the scale used in the study had been authorized. All participants signed consent forms to ensure they fully understood the study’s purpose, procedures, and potential risks. To protect participants’ privacy and ensure anonymity, no identifiable information, such as names, employee IDs, or contact information was collected. Participants also hold the right to withdraw from the study at any time.

### 2.5. Instruments

Before the formal investigation, 15 nurses were invited to an online pre-survey of the questionnaire. All participants agreed that the questionnaire items were easy to understand. Based on this feedback, no further questionnaire modifications were needed.

#### 2.5.1. Socio-demographic questionnaire.

The questionnaire was designed according to the purpose of this study, including gender, age, marital status, parental status (having children), working hours, educational level, clinical nursing, managerial status, weekly working hours, and night shifts.

#### 2.5.2. Emotional labor scale.

The scale was compiled by Hong et al. [31] and translated into Chinese by Yao et al. [32]. The Cronbach’s α coefficient of the Chinese version of the scale was 0.862, and the Cronbach’s α coefficients of the three dimensions were 0.881, 0.807, and 0.764, respectively. Three common factors were extracted via exploratory factor analysis, accounting for 61.28% of the cumulative variance. The scale included three dimensions: Emotional control effort in profession (7 items): when the perceived emotions are inconsistent with the emotions that need to be expressed, nurses actively regulate and express their true emotions. Patient-focused emotional suppression (5 items): suppress true emotions and try to adjust disharmonious emotions. Emotional pretense by norms (4 items): change their words and actions in emotional disguise to meet the professional requirements. There were 16 items in total; each was scored on a 5-point Likert scale, with 1 point representing “strongly disagree” and 5 points representing “strongly agree”. The total score ranged from 16 to 80 points. Higher scores indicate a higher level of emotional labor. We divided emotional labor into three levels based on the first and last 33.33% of the average score: low (1–2.33), medium (2.34–3.66), and high (3.67–5). In this study, the Cronbach’s α coefficient of this scale was 0.942, and the Cronbach’s α of the three dimensions were 0.964, 0.918, and 0.818, respectively. Three common factors were extracted via exploratory factor analysis, accounting for 79.34% of the cumulative variance.

#### 2.5.3. Spiritual climate scale.

The scale was compiled by Doram et al. [28] and translated and revised by Wu et al. [33]. The Cronbach’s α coefficient of the Chinese version of the scale is 0.833. One common factor was extracted via exploratory factor analysis, accounting for 66.79% of the cumulative variance. The scale is a single-dimensional, 4-item Likert scale; each item is scored on a 5-point scale from “strongly disagree” to “strongly agree,” and the total score ranges from 4 to 20. Higher scores represent a better spiritual climate of the working environment of the subjects. In this study, the Cronbach’s α coefficient of this scale was 0.960. One common factor was extracted via exploratory factor analysis, accounting for 89.58% of the cumulative variance.

#### 2.5.4. Compassion satisfaction scale.

The scale was compiled by Dr. Stamm [29], translated and modified by Chen et al. [34]. It includes three independent subscales: compassion satisfaction, secondary trauma, and burnout, with Cronbach’s α coefficients of 0.87, 0.73, and 0.84, respectively. In this study, 10 items of the compassion satisfaction subscale were selected, and the Likert 5-point scoring method was adopted, ranging from 1 (no) to 5 (always), with a total score ranging from 10 to 50 points. Higher scores represented a greater sense of happiness and achievement that participants could obtain in nursing. In this study, the Cronbach’s α coefficient of this scale was 0.969. One common factor was extracted via exploratory factor analysis, accounting for 79.87% of the cumulative variance.

### 2.6. Data analysis

IBM SPSS 26 software was used for data analysis. Examples, percentages, means, and standard deviations were used to describe general data, emotional labor, spiritual climate, and compassion satisfaction scores. T-test and one-way analysis of variance were used to examine the relationship between general data and emotional labor. A Pearson correlation analysis was used to examine the relationships among spiritual climate, compassion satisfaction, and emotional labor. Multiple linear regression analysis was used to investigate the effects of general data, spiritual climate, and compassion satisfaction on emotional labor. Power analysis was conducted with G*Power 3.1.9.7 software. Test level α = 0.05.

### 2.7. Common method deviation test

Given the potential for common method bias (CMV) in the self-reported data in this study, we conducted the survey anonymously and randomized the order of questionnaire items. The CMV was evaluated by Harman’s single-factor test. All items from the emotional labor, spiritual climate, and compassion satisfaction scales were included in the confirmatory factor analysis, and the number of common factors was set to 1. The results showed that: X^2^/df = 11.71, GFI = 0.59, RMSEA = 0.19, RMR = 0.08, CFI = 0.61, NFI = 0.59, NNFI = 0.58. None of the above indicators met the corresponding standards, indicating that CMV was not a significant concern in this study.

## 3. Results

### 3.1. Demographic and other data of study participants

Table 1 describes the general characteristics of the respondents. Most of the participants were married (78.5%) female (98.37%) between 31 and 40 years old (63.84%) with children (75.24%). Over half of them had more than 10 years of working experience (58.63%) and worked no more than 40 hours per week (61.56%). Most of them were non-managerial (82.74%) clinical nurses (90.23%) with bachelor’s degrees or above (92.51%) who had night shifts (84.36%).

**Table 1:** General information of survey subjects (n = 307).

Project	Categories	n	%
Gender	male	5	1.63
	female	302	98.37
Age	18-30 years	83	27.04
	31-40 years	196	63.84
	＞40 years	28	9.12
Marital status	unmarried	51	16.61
	married	241	78.50
	divorced	15	4.89
Children	without	76	24.76
	with	231	75.24
Working experience	≤10 years	127	41.37
	＞10 years	180	58.63
Education degree	technical secondary/college degree	23	7.49
	Bachelor or above	284	92.51
Clinical nursing	no	30	9.77
	yes	277	90.23
Managerial status	no	254	82.74
	yes	53	17.26
Working hours(weekly)	≤40h	189	61.56
	＞40h	118	38.44
Night shift	no	48	15.64
	yes	259	84.36

### 3.2. Emotional labor, spiritual climate, and compassion satisfaction of pediatric nurses

The overall emotional labor score among 307 pediatric nurses in Yichang was (69.20 ± 9.40) The item-level mean was (4.32 ± 0.59), indicating a high level of emotional labor. The total spiritual climate score was (17.13 ± 2.81), with an item-level mean of (4.28 ± 0.70). The total compassion satisfaction score was (41.71 ± 7.97), with an item-level mean of (4.17 ± 0.80). As detailed in Table 2.

**Table 2:** Current score status of pediatric nurse emotional labor, spiritual climate, and compassion satisfaction (n = 307).

Project	Entry	Score range	Overall score M(SD)	Mean scor M(SD)
emotional labor	16	16-80	69.20(9.40)	4.32(0.59)
Emotional control effort in profession	7	7-35	31.65(4.00)	4.52(0.57)
Patient-focused emotional suppression	5	5-25	21.7(3.53)	4.34(0.71)
Emotional pretense by norms	4	4-20	15.84(3.32)	3.96(0.83)
Spiritual climate	4	8-20	17.13(2.81)	4.28(0.70)
Compassion satisfaction	10	18-50	41.71(7.97)	4.17(0.80)

### 3.3. Univariate analysis of pediatric nurses’ emotional labor

Table 3 described associations between demographic and other characteristics and pediatric nurses’ emotional labor level. Scores for nurses without children (71.34 ± 8.07) were higher than those nurses with children (68.49 ± 9.71), and the difference was significant (P < 0.05). Emotional labor scores did not differ by gender, age, marital status, working experience, educational level, clinical nursing, managerial status, weekly working hours, or night shift (P > 0.05).

**Table 3:** Univariate analysis of pediatric nurses’ emotional labor (n = 307).

Project	Categories	n	Total score of EL	t/F	P
Gender	male	5	67.40(6.19)	0.431	0.667
	female	302	69.23(9.45)		
Age	18-30 years	83	70.63(8.20)	1.932	0.147
	31-40 years	196	68.41(10.05)		
	＞40 years	28	70.50(7.40)		
Marital status	unmarried	51	71.57(7.57)	2.103	0.124
	married	241	68.80(9.65)		
	divorced	15	67.47(10.12)		
Children	without	76	71.34(8.07)	2.308	0.022
	with	231	68.49(9.71)		
Working experience	≤10 years	127	69.98(9.72)	1.231	0.219
	＞10 years	180	68.64(9.15)		
Education degree	Technical secondary/college degree	23	72.87(7.97)	1.957	0.051
	bachelor	284	68.90(9.45)		
Clinical nursing	no	30	67.87(12.17)	0.817	0.415
	yes	277	69.34(9.06)		
Managerial status	no	254	68.91(9.62)	1.165	0.245
	yes	53	70.57(8.22)		
Working hours(weekly)	≤40h	189	69.39(8.74)	0.454	0.650
	＞40h	118	68.89(10.4)		
Night shift	no	48	69.85(7.75)	0.525	0.6
	yes	259	69.08(9.68)		

### 3.4. Correlation analysis of pediatric nurses’ emotional labor with spiritual climate and compassion satisfaction

Correlations among emotional labor, spiritual climate, and compassion satisfaction were expressed by Pearson’s r. Both spiritual climate and compassion satisfaction were positively associated with pediatric nurses’ emotional labor and its three dimensions (P < 0.05). As detailed in Table 4.

**Table 4:** Correlation analysis between pediatric nurses’ emotional labor and spiritual climate and compassion satisfaction (n = 307).

Project	Spiritual climate	compassion satisfaction
Emotional labor	0.669^a^	0.702^a^
Emotional control effort in profession	0.672^a^	0.720^a^
Patient-focused emotional suppression	0.648^a^	0.663^a^
Emotional pretense by norms	0.450^a^	0.502^a^

Note: a: P < 0.05

### 3.5. Multiple linear regression analysis on the influence of pediatric nurses’ emotional labor

Scatter plots were constructed with emotional labor on the y-axis and spiritual climate and compassion satisfaction on the x-axis, revealing that emotional labor and both spiritual climate and compassion satisfaction may be positively associated. Similarly, compassion satisfaction also seemed to be positively associated with emotional labor. In regression analysis, the central assumption concerned the approximate normality of residuals rather than the dependent variable; therefore, we computed standardized residuals for the four regression equations in Tables 5 and 6 and plotted their histograms to assess normality. The histograms indicated that standardized residuals conformed, or approximately conformed to a normal distribution. In addition, tests of homoscedasticity indicated that assumptions were satisfied (model 1: F = 0.072, P = 0.788; model 2: F = 2.360, P = 0.126; model 3: F = 0.646, P = 0.422; model 4: F = 1.862, P = 0.173).

**Table 5:** Multiple linear regression analysis of emotional labor among pediatric nurses (n = 307).

Factor	Emotional labor (model 1)				
	B	SE	β	p	VIF
Constant	32.230	2.524		＜0.001	
Children without ^a^					
with	−2.370	0.886	−0.109	0.008	1.001
Spiritual climate	0.877	0.193	0.262	＜0.001	2.001
compassion satisfaction	0.569	0.068	0.483	＜0.001	2.003
F	99.873				
P	0.001				
R^2^	0.497				

Note: B, unstandardized coefficients; R^2^, R-square; SE, standard error; VIF, variance inflation factor; β, standardized coefficients; ^a^: dummy reference group.

**Table 6:** Multiple linear regression analysis of emotional control effort in profession, patient-focused emotional suppression Emotional, and pretense by norms among pediatric nurses (n = 307).

Factor	Emotional control effort in profession (model 2)					Patient-focused emotional suppression (model 3)					Emotional pretence by norms (model 4)				
	B	SE	β	p	VIF	B	SE	β	p	VIF	B	SE	β	p	VIF
Constant	16.710	1.103		＜0.001		8.923	1.019		＜0.001		6.598	1.098		＜0.001	
Children without ^a^															
with	−1.150	0.387	−0.124	0.003	1.001	−0.936	0.358	−0.115	0.009	1.001	−0.284	0.386	−0.037	0.462	1.001
Spiritual climate	0.324	0.084	0.227	＜0.001	2.001	0.363	0.078	0.289	＜0.001	2.001	0.190	0.084	0.160	0.024	2.001
compassion satisfaction	0.246	0.030	0.490	＜0.001	2.003	0.174	0.027	0.394	＜0.001	2.003	0.149	0.030	0.357	＜0.001	2.003
F	89.675					72.401					31.400				
P	＜0.001					＜0.001					＜0.001				
R^2^	0.470					0.418					0.237				

Note: B, unstandardized coefficients; R^2^, R-square; SE, standard error; VIF, variance inflation factor; β, standardized coefficients; a: dummy reference group.

Tables 5 and 6 present regression analysis with demographic and other characteristics. Spiritual climate and compassion satisfaction were examined as factors associated with emotional labor in pediatric nurses. The model explained 49.7% of the variance in emotional labor at α = 0.05 (F = 99.873, P < 0.001). Parental status (having children), spiritual climate, and compassion satisfaction were significantly associated with emotional labor (P ＜ 0.05). Specifically, emotional labor among nurses with children was significantly lower than among nurses without children (β = −2.73, P = 0.008). Higher spiritual climate scores were associated with significantly higher emotional labor scores (β = 0.887, P = 0.001). Higher compassion satisfaction scores were also associated with significantly higher emotional labor scores (β = 0.569, P = 0.001).

To further understand factors associated with emotional labor, we conducted regression analyses for the three subscales of emotional labor. Table 6 describes factors associated with emotional control effort in the profession, patient-focused emotional suppression, and emotional pretense by norms. At α = 0.05 (F = 37.101, 28.077, and 33.000; all P < 0.001), 47.0%, 41.8%, and 23.7% of the variance were explained, respectively. For emotional control effort in the profession, levels were significantly higher among pediatric nurses without children (β = −1.15, SE = 0.387, P < 0.01). Spiritual climate (β = 0.324, SE = 0.084, P < 0.001) and compassion satisfaction (β = 0.246, SE = 0.03, P < 0.001) were positively associated with emotional control effort in the profession. For patient-focused emotional suppression, levels among pediatric nurses with children were significantly lower than among those without children (β = −0.936, SE = 0.358, P < 0.01). Spiritual climate (β = 0.363, SE = 0.078, P < 0.001) and compassion satisfaction (β = 0.174, SE = 0.027, P < 0.001) were both positively associated with patient-focused emotional suppression. Parental status was not significantly associated with emotional pretense in line with norms (P > 0.05). Spiritual climate (β = 0.19, SE = 0.084, P < 0.05) and compassion satisfaction (β = 0.149, SE = 0.03, P < 0.001) were positively associated with emotional pretense driven by norms.

### 3.6. Power analysis

Post hoc power analysis was conducted using G*Power 3.1.9.7 (based on the observed R^2^). For multiple linear regression, α was set as 0.05, with 3 predictor variables and a sample size of 307. Based on the observed effect sizes (R2 ranging from 0.237 to 0.497), power was > 0.999, indicating that the current sample size was sufficient to detect significant effects.

## 4. Discussion

The purpose of this study was to assess the current level of emotional labor and the factors associated with it among pediatric nurses in Yichang. The results showed that pediatric nurses in Yichang manifested a high level of emotional labor, which was associated with parental status (having children), spiritual climate, and compassion satisfaction.

### 4.1. High level of pediatric nurses’ emotional labor

Pediatric nurses in Yichang exhibited a high level of emotional labor, which was consistent with Zhang et al. [35] and Ou et al. [36], but higher than that reported in a nationwide cross-sectional survey in China by Wu et al. [37]. The pediatric nursing context is distinctive, as nurses interact with ill children and their families, more emotional involvement and regulation is required. Young pediatric patients have limited communication abilities and their physical conditions can change rapidly, and family caregivers tend to hold high expectations for nursing services. All these contextual features are associated with higher levels of emotional labor [38]. Moreover, the requirement to display patience, care, and compassion, and to maintain a positive emotional stance despite personal stress or negative emotions, is also associated with higher emotional labor levels [39]. Among the three subscales, professional emotional control effort showed the highest scores, which is consistent with Pan et al. [40]. This pattern may be related to the need to strictly adhere to professional norms. When interacting with sick children and their family caregivers, nurses often display a high degree of emotional control to maintain professional image and quality of care [41]. Such sustained regulation (remaining calm, patient, and caring in the face of children’s distress, family anxiety, and personal stress) is consistent with substantial use of emotional resources and may help explain the higher scores observed.

Emotional pretense by norms showed the lowest scores, yet it remains noteworthy. This dimension reflected adherence to institutional regulations and professional display of rules (e.g., smiling, patience) even when emotions are not fully congruent with inner states [42]. The observed distribution suggested that pediatric nurses prioritize occupational requirements and patients’ needs while aligning with social norms to some extent, which may help explain the observed differences across emotional labor dimensions.

### 4.2. The association between parental status and pediatric nurses’ emotional labor

Parental status (having children) was associated with higher emotional labor, particularly in professional emotional control effort and patient-focused emotional suppression. Within the Conservation of Resources (COR) framework [20–21], parenting experience is conceptualized as a psychological resource; it may help nurses better understand and be more compassionate toward patients. Its emotional regulation is similar to deep acting, with less resource consumption. In contrast, nurses without parenting experience may invest more cognitive and emotional effort in interpreting children’s needs, generating appropriate responses and maintaining professional images, thereby contributing to a higher emotional labor burden. In Chinese nursing culture, ideals of “selfless dedication” and “maternal role” are highly praised; the “role-resource gap” nurses face to meet such career expectations is associated with increased pressure to regulate emotions.

### 4.3. Spiritual climate is positively associated with pediatric nurses’ emotional labor

Spiritual climate was positively associated with emotional labor, which was consistent with Guo et al. [43]. Spiritual climate refers to a supportive workplace atmosphere characterized by trust, shared inner experiences, and collaborative communication [44], and it is closely linked to leadership, organizational identification, and interpersonal relationships [45]. Within the COR framework, spiritual climate is better understood as a contextual resource: supportive leadership, work guidance and supervision, emotional support, and resource provision may motivate nurses to invest more emotional resources in emotion display regulation [46]. A stronger sense of indispensability and organizational identification is associated with reduced resentment toward emotional labor and more effective emotion regulation, while colleague relationships may provide stronger emotional support, and burden-sharing may contribute to greater emotion regulation capacity [45]. Overall, spiritual climate appeared to be linked to more sustained and authentic engagement in emotional labor among pediatric nurses (more frequently associated with deep acting).

### 4.4. Compassion satisfaction is positively associated with pediatric nurses’ emotional labor

Compassion satisfaction was positively associated with emotional labor. Compassion satisfaction, defined as the positive emotional experience and professional fulfillment derived from helping patients [47], is conceptualized within the COR framework as a personal resource that may alleviate emotional strain from emotional labor and thus be associated with a reduced risk of emotional exhaustion [20]. Rather than acting as a “trigger”, compassion satisfaction may function as a supportive resource associated with sustained and more authentic engagement in emotional labor. Greater compassion satisfaction is often linked to stronger professional identity and a sense of accomplishment, which, in turn, is accompanied by confidence and composure during emotion regulation. In practice, nurses with higher compassion satisfaction may invest emotional resources more proactively to maintain their professional images and the quality of care. Their emotional regulation is more frequently associated with “deep acting”, which may be related to lower internal strain and greater perceived effectiveness. Compassion satisfaction is also associated with a stronger emotional bond with patients and alignment with patients’ affective needs [48–49]. Compared with surface acting, emotional regulation grounded in emotional resonance is associated with lower psychological conflict and greater perceived sustainability. Accordingly, compassion satisfaction can be viewed as a key personal resource associated with more integrated and less self-depleting management of professional emotional demands.

### Limitations

We used convenience sampling and recruited only pediatric nurses in Yichang. Pediatric nurses from other cities were not investigated, so there might be selection bias. For example, the number of male pediatric nurses included was relatively small. The data in this study came from nurses’ self-reports, and self-report and recall biases may have occurred. In addition, as a cross-sectional study, it cannot determine a causal relationship among demographic characteristics, spiritual climate, compassion satisfaction, and emotional labor. Finally, failure to control for confounding factors can cause residual confounding.

## 5. Conclusions

This study showed that pediatric nurses in Yichang manifested high levels of emotional labor. Parental status (having children), spiritual climate, and compassion satisfaction were significantly associated with emotional labor among pediatric nurses. Among subscales, professional emotional control effort had the highest scores, followed by patient-focused emotional suppression, and then emotional pretense by norms.

### Recommendations

Based on the findings of this study, we propose the following strategies to manage emotional labor among pediatric nurses: (1) To monitor emotional labor levels regularly to identify potential adverse outcomes associated with excessive emotional labor. (2) To improve the working environment and spiritual climate, including strengthening leadership support, promoting teamwork, and fostering positive organizational culture, which can enhance nurses’ sense of security, belonging, and motivation to some extent. (3) Prioritizing pediatric nurses’ emotional health and seeking to improve compassion satisfaction by providing psychological support, encouraging positive feedback, and delivering emotion-focused education. These approaches may reduce negative affect, improve emotional regulation, and enhance a sense of professional accomplishment. (4) To provide differentiated support for nurses with and without children, with additional emotional support and training for nurses without children to help them cope with the high intensity of emotional labor. These strategies may improve nursing quality, occupational health, and work experience among pediatric nurses.
